# First Trimester Exposure to Anxiolytic and Hypnotic Drugs and the Risks of Major Congenital Anomalies: A United Kingdom Population-Based Cohort Study

**DOI:** 10.1371/journal.pone.0100996

**Published:** 2014-06-25

**Authors:** Lu Ban, Joe West, Jack E. Gibson, Linda Fiaschi, Rachel Sokal, Pat Doyle, Richard Hubbard, Liam Smeeth, Laila J. Tata

**Affiliations:** 1 Division of Epidemiology & Public Health, Nottingham City Hospital, University of Nottingham, Nottingham, United Kingdom; 2 Department of Non-communicable Disease Epidemiology, London School of Hygiene & Tropical Medicine, London, United Kingdom; Radboud University, Netherlands

## Abstract

**Background:**

Despite their widespread use the effects of taking benzodiazepines and non-benzodiazepine hypnotics during pregnancy on the risk of major congenital anomaly (MCA) are uncertain. The objectives were to estimate absolute and relative risks of MCAs in children exposed to specific anxiolytic and hypnotic drugs taken in the first trimester of pregnancy, compared with children of mothers with depression and/or anxiety but not treated with medication and children of mothers without diagnosed mental illness during pregnancy.

**Methods:**

We identified singleton children born to women aged 15–45 years between 1990 and 2010 from a large United Kingdom primary care database. We calculated absolute risks of MCAs for children with first trimester exposures of different anxiolytic and hypnotic drugs and used logistic regression with a generalised estimating equation to compare risks adjusted for year of childbirth, maternal age, smoking, body mass index, and socioeconomic status.

**Results:**

Overall MCA prevalence was 2.7% in 1,159 children of mothers prescribed diazepam, 2.9% in 379 children with temazepam, 2.5% in 406 children with zopiclone, and 2.7% in 19,193 children whose mothers had diagnosed depression and/or anxiety but no first trimester drug exposures. When compared with 2.7% in 351,785 children with no diagnosed depression/anxiety nor medication use, the adjusted odds ratios were 1.02 (99% confidence interval 0.63–1.64) for diazepam, 1.07 (0.49–2.37) for temazepam, 0.96 (0.42–2.20) for zopiclone and 1.27 (0.43–3.75) for other anxiolytic/hypnotic drugs and 1.01 (0.90–1.14) for un-medicated depression/anxiety. Risks of system-specific MCAs were generally similar in children exposed and not exposed to such medications.

**Conclusions:**

We found no evidence for an increase in MCAs in children exposed to benzodiazepines and non-benzodiazepine hypnotics in the first trimester of pregnancy. These findings suggest that prescription of these drugs during early pregnancy may be safe in terms of MCA risk, but findings from other studies are required before safety can be confirmed.

## Introduction

Mental illness is among the leading causes of disability in the United Kingdom (UK) [1]. Compared with men, women are more likely to develop common mental disorders such as depression and anxiety [2], which often require drug treatment. About 3% of women are prescribed anxiolytics, hypnotics, or antidepressants around early pregnancy in the UK [3], a similar level to those in other countries [4]. Since the Thalidomide scandal [5], the potential for teratogenic effects of drugs has been a pressing concern for all women of childbearing age and prescribers. Due to their widespread use there has rightly been a research focus on maternal perinatal mental health [6] and its potential effects on children born to women taking antidepressants, mood stabilisers and antipsychotic drugs [7]. By contrast, research into anxiolytic and hypnotic drugs, which are commonly prescribed in association with anxiety symptoms is severely lacking in both quantity and quality [8]. Very few studies have examined the effect of individual drugs or assessed the impacts of underlying health conditions and concurrent medications [8].

A 2011 meta-analysis [9] showed no association between congenital anomalies overall and benzodiazepine exposure in pregnancy, however it was not a systematic review with any formal assessment of the quality of included studies. In this analysis there was marked heterogeneity between studies, the results were mostly driven by two studies [10], [11], and different abnormalities were included in each. Previous studies have seldom distinguished between benzodiazepines and non-benzodiazepine hypnotics (i.e. zopiclone, zaleplone and zolpidem), which differ in chemical structure. A population-based study from Sweden published in the same year [12] showed little evidence for the teratogenicity of non-benzodiazepine hypnotics, although they had previously [10] found a 1.4-fold increased risk of major congenital anomalies (MCAs) associated with antenatal exposure to benzodiazepines. Evidence for system-specific congenital anomalies is even more limited. Early case-control studies reported increased risks of facial clefts with benzodiazepines [13], [14], which have not been found in more recent research [15]–[17]. Only four studies so far have been conducted to investigate the risk of heart anomalies and have not consistently shown increased risks [11], [18]–[20]. In addition, a study from British Columbia [11] highlighted the combined effect of taking both antidepressants and benzodiazepines and suggested that such dual drug exposure, rather than benzodiazepines alone, was associated with an increased risk of congenital heart anomalies. Such concurrent exposure is common and may not have been accounted for in many other studies.

We have therefore conducted the first UK population-based study using routinely-collected primary care data to investigate whether first trimester exposure to benzodiazepine anxiolytics/hypnotics and non-benzodiazepine hypnotics without concurrent antidepressant exposure increases the risk of MCAs. We estimated such risks for system-specific congenital anomalies and the comparative risks among children born to women with depression or anxiety but with no first trimester psychotropic medication.

## Methods

### Ethics Statement

All data are anonymised, such that individual patients as well as the name and specific location of general practices cannot be identified by researchers. Ethical approval for this study was obtained from the Medical Research Ethics Committee (administered and approved by the National Health Service South East Research Ethics Committee) REC reference 04/MRE01/9.

### Study population

We used a pregnancy cohort study design which included all singleton live births for women aged 15–45 years between 1990 and 2010 from The Health Improvement Network (THIN), where anonymised children's and mothers' medical records were linked to provide prospectively recorded information before, during and after pregnancy. THIN is a nationally representative database of computerised longitudinal general practice records of prospectively-collected health information across the UK. The UK's National Health Service (NHS) is tax-payer funded and provides access to all basic health care free at source including essential drug prescriptions at a minimal charge. As part of the NHS, general practice (primary health care units) is responsible for overseeing patients' medical care which includes coordination of their health care from hospital or other secondary care services and is the first point of contact for non-emergency access to almost all national health care services. These data are therefore primarily collected and recorded for the purpose of routine management of patient health care in the UK NHS general practice setting, rather than for research purposes. The version of THIN used for the purpose of this study contained records from 495 general practices throughout the UK, covering 5% of the total UK population. THIN contains valid medical diagnoses, events, symptoms and drug prescriptions and is widely used for pharmacoepidemiological studies [21]. Prescriptions are automatically entered at the point of issue on the database such that error in their measurement will be minimal. Whilst medications can also be prescribed in hospital, the vast majority are prescribed via the patient's general practice, in particular if they are repeat prescriptions. Since treatment for anxiety and depression is almost entirely managed in general practice, it is unlikely that the medications assessed in this study will be prescribed in hospital and none are available without a doctor's prescription in the UK. Furthermore, during pregnancy women receive free prescriptions via their general practice so it is unlikely that we are missing prescriptions.

We excluded women with serious mental illness (i.e. bipolar disorder, schizophrenia and other related psychotic disorders) and women with epilepsy diagnoses or with prescriptions of antiepileptic drugs in pregnancy (4,739 pregnancies/1.2% of the total population) since previous literature has shown increases of congenital anomalies in children born to women treated for such conditions [22]–[24].

### Outcome definitions

We extracted all diagnostic recordings of MCAs (excluding genetic anomalies and anomalies attributed to known teratogens, e.g. anomalies due to maternal infections and fetal alcohol syndrome) from the children's general practice records and classified these into system-specific groups by using Read codes corresponding to the European Surveillance of Congenital Anomalies classification [25], which is based on the International Classification of Disease (ICD-10) [26]. The recording of MCAs among live births in THIN have shown to be highly comparable to UK national registry data that contribute the EUROCAT [26]. Furthermore, the recording of MCAs in general practice data have shown to have good specificity when validated against medical notes [27]–[30]. Routine general practice data has also been shown to be a useful source to monitor the outcomes of pregnancies exposed to common environmental and medical teratogens [31], [32].

### Exposure definitions

We identified all benzodiazepine drugs (diazepam, alprazolam, chlordiazepoxide hydrochloride, lorazepam, oxazepam, nitrazepam, flurazepam, loprazolam, lormetazepam, temazepam, clonazepam, clobazam and triazolam) and non-benzodiazepine hypnotics (zopiclone, zaleplone and zolpidem) prescribed in UK primary care. In the UK, these drugs are not available as over the counter drugs and can only be issued with a prescription from a health care professional. Antenatal exposure to these drugs during the first trimester of pregnancy was defined according to the presence or absence of a relevant drug prescription in women's primary care electronic health records from four weeks before the estimated onset of the last menstrual period up to 12 weeks after so as to include drug prescriptions received immediately before pregnancy and potentially used during early pregnancy. Dates of onset of the last menstrual period were estimated based on a range of recordings of information related to women's pregnancy, delivery and gestational age of their children, and where no information was available, live births were assumed to take place at 40 weeks.

Nearly 90% of children whose mothers were prescribed such drugs in this period (2,904 out of 3,218) were exposed to diazepam, temazepam or zopiclone. For the remaining 314 children exposed to benzodiazepines and non-benzodiazepine hypnotics other than these three we had very few exposed cases to examine each drug separately and therefore grouped them together. In addition, diagnostic recordings of depression and anxiety (including generalised anxiety disorder, panic attacks, insomnia and other anxiety related disorder) in the year before pregnancy or during pregnancy were also identified from women's medical records. The prevalence estimates of maternal depression and anxiety in and around pregnancy in THIN are similar to studies using standardised clinical interviewing schedules [33].

First-trimester exposure to all antidepressants, including tricyclic antidepressants, selective serotonin reuptake inhibitors (SSRIs) and other antidepressants in the British National Formulary 4.3 [34] were also extracted from women's medical records. Since we primarily aimed to assess the MCA risk for exposure to benzodiazepines and non-benzodiazepine hypnotics with/without concurrent exposure to antidepressants, children born to women with prescriptions of an antidepressant but no anxiolytic/hypnotic drugs in the first trimester were excluded from the study (3.2% of the total population; 12,399 pregnancies). Therefore, based on mothers' exposure status, we classified children into 10 antenatal exposure groups: 1) mothers with no diagnoses of depression or anxiety (the baseline group), 2) mothers with diagnosed depression or anxiety in the year before pregnancy or during pregnancy but without anxiolytic, hypnotic or antidepressant drugs deemed above as first trimester exposures, 3–6) mothers prescribed diazepam, temazepam, zopiclone or other anxiolytic/hypnotic drugs without concurrent prescriptions of antidepressants, 7–10) mothers prescribed diazepam, temazepam, zopiclone or other anxiolytic/hypnotic drugs with co-prescriptions for antidepressants in the first trimester. Children of mothers exposed to more than one type of anxiolytic/hypnotic drugs were included in multiple groups.

### Covariates

For each pregnancy, we also extracted the year of the child's birth, maternal age at childbirth and socioeconomic status as measured by quintiles of the Townsend Index of Deprivation [35]. In addition, we extracted the most recent maternal smoking status before delivery and used a previously validated algorithm to classify women's smoking status as never smoking, ex-smoking, and current smoking [36], [37]. We also extracted the most recent maternal body mass index (BMI) measurement before pregnancy and classified as normal, underweight, overweight and obese according to the WHO classification [38].

### Statistical Analysis

To estimate the disease burden of all MCAs and across 14 system-specific groups we calculated absolute risks (per 10,000 live births) for each antenatal exposure group. Multiple logistic regression with generalised estimating equation modelling [39], to account for potential clustering effects of children born to the same woman in consecutive pregnancies, was used to estimate odds ratios (ORs) with 99% confidence intervals (CIs) for the associations of any MCA (and the three most prevalent system-specific groups: heart, limbs and genital system) with each exposure group. We adjusted the results for the year of the child's birth, maternal age at childbirth, socioeconomic status, smoking and BMI. Children of mothers with missing information on smoking or BMI were included in separate categories in the multivariate analysis. The same analyses were repeated using children of women with un-medicated depression/anxiety (group 2) as the baseline group.

### Sensitivity analyses

We conducted three sensitivity analyses to ensure the robustness of the study results. Firstly, we restricted the group of children whose mothers were prescribed benzodiazepines or non-benzodiazepine hypnotics to those of mothers with monotherapy only and compared the risks of overall MCA and the three most prevalent system-specific groups to the risks in children of mothers without depression or anxiety. Secondly, we repeated the main analysis after restricting the drug-exposed groups to those children born to women with at least one high-dose prescription. The high-dose prescription was defined as 15 mg or above per day for diazepam, 20 mg or above per day for temazepam, and 7.5 mg or above per day for zopiclone. Thirdly, we repeated the main analysis after restricting the drug-exposed groups to those children born to women with at least two prescriptions for the same individual drug. All analyses were carried out using Stata SE 11.0 (Stata Corp., College Station, TX, USA).

### Power calculation

Based on the study population we used for the purpose of this study, we estimated that we had over 90% power to detect an OR of 2.0 for the association of MCA overall with antenatal exposure to un-medicated depression/anxiety or with exposure to diazepam in the first trimester at 1% significance level (99% CIs). We had in contrast 60% power to detect a similar effect for temazepam and zopiclone. However, when we changed the significance level to 5% (95% CIs), we had nearly 80% power to detect a similar effect for temazepam and zopiclone. This power calculation was performed using G*Power 3.1 [40].

## Results

Of 374,196 live-born singletons, 2.7% (99%CI 2.6–2.7%) had major congenital anomalies. The median maternal age at birth was 29 years (interquartile range 25–33). Children with MCAs had similar maternal characteristics to children without MCAs (Table 1). There were 19,193 (5.1%) children born to women with diagnosed depression or anxiety but with no first trimester medication and 3,218 (0.9%) with first trimester exposure to anxiolytic or hypnotic drugs, of which 1,175 children (36.5%) had concurrent exposure to antidepressants (65.5% of which were SSRIs). Women prescribed anxiolytic/hypnotic drugs were more likely to be from socioeconomically deprived groups than women with depression/anxiety un-medicated in early pregnancy (Table 2).

**Table 1:** Maternal characteristic of children with and without major congenital anomalies.

	All children		Children without MCAs		Children with MCAs	
	N = 374,196		n = 364,214		n = 9,982 [2.7%]	
	n	*%*	n	*%*	n	*%*
**Maternal age** at the end of pregnancy, years (Median, IQR)	29	*25–33*	29	*25–33*	29	*25–33*
**Townsend deprivation index**						
1 (Least deprived)	90,671	24.2	88,200	24.2	2,471	24.8
2	71,478	19.1	69,596	19.1	1,882	18.9
3	72,020	19.2	70,071	19.2	1,949	19.5
4	67,115	17.9	65,360	17.9	1,755	17.6
5 (Most deprived)	49,797	13.3	48,438	13.3	1,359	13.6
Missing	23,115	6.2	22,549	6.2	566	5.7
**Maternal smoking status**						
Never	101,006	27.0	98,465	27.0	2,541	25.5
Current smoker	52,348	14.0	51,000	14.0	1,348	13.5
Ex-smoker	124,960	33.4	121,530	33.4	3,430	34.4
Missing	95,882	25.6	93,219	25.6	2,663	26.7
**Maternal BMI** (kg/m^2^)						
Normal (18.5–24.9)	160,544	42.9	156,382	42.9	4,162	41.7
Underweight (<18.5)	30,384	8.1	29,597	8.1	787	7.9
Overweight (25–29.9)	59,267	15.8	57,693	15.8	1,574	15.8
Obese (> = 30)	32,132	8.6	31,209	8.6	923	9.2
Missing	91,869	24.6	89,333	24.5	2,536	25.4

MCAs = major congenital anomalies; BMI = body mass index.

**Table 2:** Maternal characteristics of children born to women with and without benzodiazepine prescriptions during the first trimester of pregnancy.

	Baseline group without depression or anxiety		Depression or anxiety without drug exposures		Diazepam^a^		Temazepam^a^		Zopiclone^a^	
	n = 351,785 [94.0%]		n = 19,193 [5.1%]		n = 1,159 [0.31%]		n = 379 [0.10%]		n = 406 [0.11%]	
	n	*%*	n	*%*	n	*%*	n	*%*	n	*%*
**Maternal age** at the end of pregnancy, years (Median, IQR)	29	*25–33*	28	*24–33*	30	*26–34*	29	*25–33*	30	*25–34*
**Townsend deprivation index**										
1 (Least deprived)	86,640	*24.6*	3,505	*18.3*	229	*19.8*	64	*16.9*	68	*16.7*
2	67,934	*19.3*	3,093	*16.1*	184	*15.9*	69	*18.2*	53	*13.1*
3	67,723	*19.3*	3,705	*19.3*	218	*18.8*	69	*18.2*	74	*18.2*
4	62,188	*17.7*	4,189	*21.8*	238	*20.5*	80	*21.1*	88	*21.7*
5 (Most deprived)	45,660	*13.0*	3,460	*18.0*	204	*17.6*	74	*19.5*	91	*22.4*
Missing	21,640	*6.2*	1,241	*6.5*	86	*7.4*	23	*6.1*	32	*7.9*
**Maternal smoking status**										
Never	96,368	*27.4*	3,977	*20.7*	268	*23.1*	78	*20.6*	93	*22.9*
Current smoker	47,083	*13.4*	4,287	*22.3*	287	*24.8*	84	*22.2*	140	*34.5*
Ex-smoker	118,210	*33.6*	5,934	*30.9*	334	*28.8*	100	*26.4*	95	*23.4*
Missing	90,124	*25.6*	4,995	*26.0*	270	*23.3*	117	*30.9*	78	*19.2*
**Maternal BMI** (kg/m^2^)										
Normal (18.5–24.9)	150,910	*42.9*	8,286	*43.2*	500	*43.1*	161	*42.5*	152	*37.4*
Underweight (<18.5)	28,178	*8.0*	1,870	*9.7*	114	*9.8*	41	*10.8*	48	*11.8*
Overweight (25–29.9)	55,309	*15.7*	3,349	*17.4*	207	*17.9*	59	*15.6*	95	*23.4*
Obese (> = 30)	29,573	*8.4*	2,170	*11.3*	133	*11.5*	40	*10.6*	51	*12.6*
Missing	87,815	*25.0*	3,518	*18.3*	205	*17.7*	78	*20.6*	60	*14.8*

a Does not include children born to women with co-prescriptions of antidepressants in the first trimester of pregnancy.

The prevalence of MCA was similar across all exposure groups: 2.7% (99%CI 2.6-.27%) in the baseline group of children whose mothers were not diagnosed with depression or anxiety, 2.7% (2.4–3.0%) in those whose mothers had diagnosed depression or anxiety un-medicated in the first trimester, 2.7% (1.6–4.1%) in children exposed to diazepam without antidepressants in the first trimester, 2.9% (1.1–5.9%) in children exposed to temazepam without antidepressants, 2.5% (0.9–5.2%) in children exposed to zopiclone without antidepressants (Table 3) and 3.4% (0.9–8.8%) in children exposed to other anxiolytic/hypnotic drugs without antidepressants. Compared with the baseline group, adjusted ORs were 1.02 (99%CI 0.63–1.64) for diazepam, 1.07 (0.49–2.37) for temazepam, 0.96 (0.42–2.20) for zopiclone (Table 4) and 1.27 (0.43–3.75) for other anxiolytic/hypnotic drugs when not concurrently prescribed an antidepressant. When assessing the MCA risk for exposure to benzodiazepines and non-benzodiazepines concurrent with antidepressants, the adjusted ORs were 1.07 (0.53–2.17) for diazepam, 1.13 (0.41–3.07) for temazepam, 1.44 (0.72–2.91) for zopiclone (Table 4) and 1.35 (0.45–4.03) for other anxiolytic/hypnotic drugs.

**Table 3:** Absolute risks (per 10,000 children) of major congenital anomalies in children born to women with and without benzodiazepine prescriptions during the first trimester of pregnancy.

	Baseline group without depression or anxiety		Depression or anxiety without drug exposures		Diazepam^a^		Temazepam^a^		Zopiclone^a^	
	n = 351,785		n = 19,193		n = 1,159		n = 379		n = 406	
	n	*risk* c	n	*risk* c	n	*risk* c	n	*risk* c	n	*risk* c
**Any MCAs**	9,368	*266*	518	*270*	31	*267*	11	*290*	10	*246*
Heart	2,642	*75*	149	*78*	12	*104*	4	*106*	6	*148*
Limb	1,833	*52*	77	*40*	6	*52*	1	*26*	1	*25*
Genital system	1,361	*39*	85	*44*	3	*26*	1	*26*	0	*0*
Urinary system	878	*25*	60	*31*	6	*52*	1	*26*	0	*0*
Chromosomal	619	*18*	32	*17*	1	*9*	0	*0*	1	*25*
Nervous system	517	*15*	31	*16*	1	*9*	2	*53*	1	*25*
Oro-facial cleft	464	*13*	35	*18*	0	*0*	0	*0*	0	*0*
Musculoskeletal system	446	*13*	27	*14*	0	*0*	1	*26*	2	*49*
Digestive system	354	*10*	17	*9*	0	*0*	0	*0*	0	*0*
Eye	342	*10*	19	*10*	2	*17*	0	*0*	0	*0*
Other CAs^b^	322	*9*	22	*11*	0	*0*	0	*0*	0	*0*
Respiratory system	219	*6*	13	*7*	0	*0*	0	*0*	1	*25*
Ear, face and neck	92	*3*	7	*4*	0	*0*	0	*0*	0	*0*
Abdominal wall	82	*2*	4	*2*	0	*0*	1	*26*	0	*0*

a Does not include children born to women with co-prescriptions of antidepressants in the first trimester of pregnancy.

b e.g. asplenia, situs inversus and skin disorders.

c per 10,000 children.

MCAs = major congenital anomalies.

**Table 4:** Association between benzodiazepine prescribing in the first trimester of pregnancy and the risk of major congenital anomalies in offspring^a^.

	Depression or anxiety without drug exposures		Diazepam^b^		Temazepam^b^		Zopiclone^b^	
	n = 19,193		n = 1,159		n = 379		n = 406	
	AOR	99%CI	AOR	99%CI	AOR	99%CI	AOR	99%CI
**Any MCAs**	1.01	0.90–1.14	1.02	0.63–1.64	1.07	0.49–2.37	0.96	0.42–2.20
Heart	1.02	0.82–1.28	1.34	0.63–2.86	1.40	0.39–5.05	1.93	0.66–5.66
Limb	0.79	0.58–1.06	1.05	0.36–3.03	0.49	0.04–6.57	0.52	0.04–6.89
Genital system	1.16	0.87–1.55	0.69	0.16–3.09	0.71	0.06–8.66	—	

a Children born to women without depression or anxiety as the comparison group & gaps in the table indicated insufficient data available for the specific comparisons.

b Does not include children born to women with co-prescriptions of antidepressants in the first trimester of pregnancy.

AOR = odds ratio adjusted for maternal age at childbirth, calendar year at birth at a categorical variables, maternal smoking, body mass index and socioeconomic status.

MCAs = major congenital anomalies; CI = confidence interval.

Absolute risks of system-specific congenital anomalies showed small variations across different exposure groups with both increases and decreases and no specific rise for children with drug exposures (Table 3). Congenital heart anomaly was the most common system-specific anomaly with an absolute risk prevalence of 0.8% in children born to women both with and without depression/anxiety, ranging from 0.9–1.7% in children of mothers prescribed diazepam, temazepam, zopiclone or other anxiolytic/hypnotic drugs without antidepressants in early pregnancy (Table 3). None of the AOR provided evidence for statistically significant differences between the exposure groups (Table 4). The absolute risks of limb and genital system anomalies were similar in children of women with and without early antenatal drug exposures (Table 3). In adjusted analyses, 99%CIs all included the null for congenital anomalies of the limbs and genital systems (Table 4).

### Results from using children born to women with un-medicated illness as the baseline group

Similar results were found when using children of mothers with diagnosed anxiety or depression but without any drug exposure in the first trimester as the baseline group (Table 5) and the adjusted ORs were 0.99 (99%CI 0.61–1.61) for diazepam, 1.04 (0.47–2.32) for temazepam, 0.93 (0.40–2.15) for zopiclone and 1.25 (0.42–3.68) for other anxiolytic/hypnotic drugs.

**Table 5:** Association between benzodiazepine prescribing in the first trimester of pregnancy and the risk of major congenital anomalies in offspring^a^.

	Diazepam^b^		Temazepam^b^		Zopiclone^b^	
	n = 1,159		n = 379		n = 406	
	AOR	99%CI	AOR	99%CI	AOR	99%CI
**Any MCAs**	0.99	0.61–1.61	1.04	0.47–2.32	0.93	0.40–2.15
Heart	1.29	0.60–2.80	1.31	0.35–4.92	2.03	0.69–6.02
Limb	1.42	0.47–4.31	—		0.64	0.05–8.49
Genital system	0.57	0.12–2.60	*—*		—	

a Children born to women with depression or anxiety but without anxiolytic, hypnotic or antidepressant drugs in the first trimester as the comparison group & gaps in the table indicated insufficient data available for the specific comparisons.

b Does not include children born to women with co-prescriptions of antidepressants in the first trimester of pregnancy.

AOR = odds ratio adjusted for maternal age at childbirth and calendar year at birth at a categorical variables, body mass index and maternal smoking and socioeconomic status.

MCAs = major congenital anomalies; CI = confidence interval.

### Results from sensitivity analyses

Table 6 shows the results from the three sensitivity analyses. After restricting our analysis to children of mothers with monotherapy only, we found almost identical results to the main analysis. There were only 379 children born to women prescribed high-dose diazepam in the first trimester, 117 for temazepam and 241 for zopiclone. Similarly, there were very few children of mothers with at least two prescriptions for the same individual drug (214 for diazepam, 55 for temazepam and 73 for zopiclone). Although the power was inevitably reduced for the latter two analyses, we found similar effect measurements to the main analysis and all 99%CIs were overlapping.

**Table 6:** Sensitivity analyses with different definitions of drug exposures.

	Diazepam^b^				Temazepam^b^				Zopiclone^b^			
	n	*risk*	AOR	99%CI	n	*risk*	AOR	99%CI	n	*risk*	AOR	99%CI
**Monotherapy only** c	**n of exposed = 1,088**				**n of exposed = 332**				**n of exposed = 366**			
**Any MCAs**	31	*285*	1.09	0.67–1.75	11	*331*	1.23	0.55–2.71	9	*246*	0.96	0.40–2.29
**Heart**	12	*110*	1.43	0.67–3.05	4	*120*	1.60	0.44–5.78	5	*137*	1.78	0.54–5.80
**Limb**	6	*55*	1.11	0.38–3.22	1	*30*	0.55	0.04–7.51	1	*27*	0.58	0.04–7.65
**Genital system**	3	*28*	0.74	0.16–3.29	1	*30*	0.80	0.07–9.85	0	*0*	-	
**High-dose only** d	**n of exposed = 379**				**n of exposed = 117**				**n of exposed = 241**			
**Any MCAs**	9	*237*	0.91	0.38–2.18	1	*85*	0.32	0.03–3.90	7	*290*	1.13	0.42–3.03
**Heart**	2	*53*	0.69	0.11–4.23	0	*0*	-		4	*166*	2.16	0.58–8.06
**Limb**	3	*79*	1.63	0.36–7.27	0	*0*	-		1	*41*	0.87	0.07–11.71
**Genital system**	1	*26*	0.73	0.06–9.04	0	*0*	-		0	*0*	-	
**Two prescriptions** e	**n of exposed = 214**				**n of exposed = 55**				**n of exposed = 73**			
**Any MCAs**	5	*234*	0.86	0.26–2.83	0	*0*	-		1	*127*	0.50	0.04–6.37
**Heart**	2	*93*	1.16	0.18–7.49	0	*0*	-		0	*0*	-	

Association between benzodiazepine prescribing in the first trimester of pregnancy and the risk of major congenital anomalies in offspring^a^.

a Children born to women without depression or anxiety as the comparison group & gaps in the table indicated insufficient data available for the specific comparisons.

b Does not include children born to women with co-prescriptions of antidepressants in the first trimester of pregnancy.

c Children born to women prescribed diazepam, temazepam, or zopiclone without other benzodiazepine or non-benzodiazepine hypnotics.

d Children born to women with at least one high-dose prescription (defined as 15 mg or above per day for diazepam, 20 mg or above per day for temazepam and 7.5 mg or above per day for zopiclone).

e Children born to women with at least two prescriptions of each individual drug.

AOR = odds ratio adjusted for maternal age at childbirth, calendar year at birth at a categorical variables, maternal smoking, body mass index and socioeconomic status.

MCAs = major congenital anomalies; CI = confidence interval.

## Discussion

### Principal Findings

We did not find that early antenatal prescriptions of diazepam, temazepam, zopiclone, or other anxiolytic/hypnotic drugs were associated with excess risks of MCA overall or with system-specific groups. The overall MCA risk was similar in children whose mothers had and had not co-prescriptions of antidepressants in the first trimester. The estimates remained almost unchanged when using children born to women with diagnosed depression or anxiety but without anxiolytic, hypnotic or antidepressant exposures as the comparison group.

### Strength and Limitations

Our study is among only a few to have estimated the risks of overall MCA and system-specific anomalies in children exposed to benzodiazepine drugs and non-benzodiazepine hypnotics excluding women who may have also been exposed to antidepressants in the first trimester of pregnancy. The exposure, outcome and covariate data were from UK general practices and recorded prospectively in the course of routine clinical care, thus excluding recall bias. Since there are a large number of comparisons in our study, we used 99%CIs to minimise the potential risk of false positive results due to multiple testing. We also adjusted for year of childbirth, maternal age at childbirth, smoking, BMI and socioeconomic status to minimise potential confounding effects from these factors.

MCA prevalence estimates across all system-specific groups and specific MCA diagnoses in the general practice database we have used have been compared with those reported in UK registers of the European Surveillance of Congenital Anomalies network, and were shown to be highly complete and specific [26]. We included MCAs diagnosed up to age 20 years where available, so we expect to have captured these outcomes as completely as registry data if not more so [26]. As stillbirths are recorded in the mother's record, but stillborn children do not have their own registration in the primary care database, we only included live-born children, as has been the case in most previous studies of congenital anomaly risk. Since stillbirth overall occurs in less than 1% of all births [41] and only 8–14% of stillbirths are believed to be due to congenital abnormalities [42], [43], the effect of excluding stillbirths on our risk estimates will be minimal.

In the UK, general practice has a gate-keeping role and is the first point of contact for non-emergency access to almost all national health care services. Moreover, all pregnant women in the UK are required to be registered with a general practitioner in order to benefit from antenatal care and free prescriptions. It is therefore unlikely that we did not identify women with prescriptions for the psychotropic drugs assessed. However, there could be potential misclassification in the exposure if a woman receiving a drug prescription did not actually take the medication or did not take it during the organogenesis period, which could bias our estimates towards a null finding. All population-based studies large enough to assess MCA risks are limited in their ability to obtain women's actual medication consumption and self-reported use has not been shown to be more reliable.

Potential confounding by indication is a limitation of all observational studies of drug safety. Because benzodiazepines are prescribed for a range of indications we excluded children whose mothers had been diagnosed with severe mental illness or epilepsy as these may have introduced important confounding. Temazepam and zopiclone are primarily indicated for insomnia which is often associated with anxiety, and diazepam is also commonly prescribed for anxiety. In this population of pregnant women, we believe that these will account for the vast majority of indications for such prescriptions. Hyperemesis gravidarum is rarely treated with diazepam in the UK and this occurs among only 0.5–2% of pregnant women [44]. Our findings that risks were similar when compared to women in the general population and women with diagnosed but un-medicated depression or anxiety are reassuring. The prevalence of diagnosed maternal depression and anxiety in UK primary care has been previously measured and shows similar estimates to survey studies using clinical diagnostic criteria [33].

### Interpretation in context of previous studies

Very few population-based studies have examined the potential teratogenic effects of individual benzodiazepines or non-benzodiazepine hypnotic drugs [8]. Two matched case-control studies using a large population-based database from the Hungarian Case-Control Surveillance of Congenital Abnormalities generally found no increased risks of congenital anomalies overall in children born to women treated with diazepam in early pregnancy [45], [46] apart from some small increases of specific anomalies such as congenital limb anomalies. The information on antenatal exposure however was partly collected through women's self-report after childbirth which is inevitably subjected to recall bias.

Although a considerable proportion of women on such medications are also prescribed other psychotropic medications such as antidepressants previous research generally has investigated the teratogenic effects of anxiolytic and hypnotic drugs without assessing concurrent medication use. A population-based study using the Swedish Medical Birth Register [10] found a 37% increased risk of MCA in children with early antenatal exposure to any benzodiazepines (OR = 1.37, 95%CI 1.07–1.76), but not to non-benzodiazepine hypnotics (OR = 1.09, 95%CI 0.68–1.75) after adjusting for some maternal factors. The authors found increases of alimentary tract atresia and pyloric stenosis (risk ratios (RR) = 2.63 and 3.80, 95%CIs 1.01–5.42 and 1.53–7.84) in children exposed to any benzodiazepines or non-benzodiazepine hypnotics in early pregnancy. However, 31% of women exposed to benzodiazepine anxiolytics or non-benzodiazepine hypnotics were also prescribed antidepressants and the authors noted that some of their benzodiazepine-exposed congenital anomaly cases were also exposed to antidepressants or anticonvulsive drugs in early pregnancy. A later study conducted by the same author [12] also found no association of maternal use of non-benzodiazepine hypnotics with relatively severe congenital anomalies overall (RR = 1.02, 95%CI 0.75–1.38) except for a five-fold increase of intestinal abnormalities other than atresia/stenosis (RR = 5.06, 95%CI 1.38–13.0). Neither of the studies however excluded use of antidepressants or anticonvulsants from their analyses.

Oberlander and colleagues, using population-based registry data from British Columbia, Canada in 2008 [11] did assess both sole- and co-prescribing and found that antenatal exposure to benzodiazepines combined with antidepressants, but not benzodiazepines alone, were associated with a higher risk of congenital heart anomalies (risk difference = 1.18, 95%CI 0.18–2.18 for dual exposure and −0.13, −0.55–0.29 for sole exposure). This Canadian study [11] however did not assess the potential contribution of underlying illnesses. It is possible that women with both antidepressant and benzodiazepine medications have more severe mental illness. Although we were unable to separate the drug effect from the underlying mental illness, we found similar effect measurements when comparing the risks of congenital anomalies in children with different anxiolytic and hypnotic drug exposures to children of mothers with depression or anxiety but without such drug treatment. In addition, since there is an increasing interest in the potential teratogenic effect of antidepressants in both research and medical practice settings, it is possible that children with dual exposure to specific antidepressants may be more likely to be assessed and subsequently diagnosed with congenital anomalies at earlier age than children of healthy mothers.

## Conclusions

This first UK population-based study found no evidence of increased risks of MCAs associated with antenatal drug exposure to diazepam, temazepam, zopiclone or other anxiolytic/hypnotic drugs. Our results indicate that prescription of these drugs during early pregnancy may be safe in terms of MCA risk, but findings from other studies are required before safety can be confirmed.
